# Selenomethionine Supplementation Mitigates Liver Dysfunction, Oxidative Injury and Apoptosis through Enhancing Antioxidant Capacity and Inhibiting JNK MAPK Pathway in Piglets Fed Deoxynivalenol-Contaminated Diets

**DOI:** 10.3390/antiox13030295

**Published:** 2024-02-28

**Authors:** Haopeng Zhong, Zhouyin Huang, Lin Li, Xingping Chen, Tiande Zou, Jun Chen, Jinming You

**Affiliations:** Jiangxi Province Key Laboratory of Animal Nutrition, College of Animal Science and Technology, Jiangxi Agricultural University, Nanchang 330045, China; zhonghaopeng@stu.jxau.edu.cn (H.Z.); hzy11373x@stu.jxau.edu.cn (Z.H.); li_lin1011@jxau.edu.cn (L.L.); cxp0315@jxau.edu.cn (X.C.); tiandezou@jxau.edu.cn (T.Z.)

**Keywords:** deoxynivalenol, JNK MAPK, liver, oxidative injury, piglets, selenomethionine

## Abstract

This research evaluated the impacts of selenomethionine (Se-Met) on hepatic functions, oxidative stress, mitochondrial function, and apoptosis of piglets fed deoxynivalenol (DON)-contaminated diets. Twenty-four piglets were allocated four dietary treatments (n = 6) in a 28-day feeding trial. The four treatments included the control group, which received 0.3 mg/kg of Se (as Se-Met) without DON treatment, and the DON treatment groups received 0, 0.3, or 0.5 mg/kg Se as Se-Met. A dietary addition of 0.5 mg/kg Se improved liver pathology and reduced serum aspartate aminotransferase and lactate dehydrogenase levels in piglets fed DON-contaminated diets. Furthermore, 0.5 mg/kg Se mitigated the oxidative stress and apoptosis of piglets fed DON-contaminated diets, as indicated by the decreased reactive oxygen species level, and the down-regulated mRNA levels of *NRF-1*, *Bax,* and *CASP9* in the liver. Importantly, 0.5 mg/kg Se enhanced the hepatic antioxidant capacity, as evidenced by increased hepatic total antioxidant capacity, catalase, glutathione peroxidase, and total superoxide dismutase activities, as well as the up-regulated mRNA levels of *Nrf2*, *Gclm*, *NQO1*, *SOD1*, and *GPX1* in the liver. Moreover, 0.5 mg/kg Se down-regulated the p-JNK protein level in the liver of piglets fed DON-contaminated diets. Collectively, Se-Met supplementation mitigated liver dysfunction, oxidative injury, and apoptosis through enhancing antioxidant capacity and inhibiting the JNK MAPK pathway in piglets fed DON-contaminated diets.

## 1. Introduction

Deoxynivalenol (DON) is a prevalent *Fusarium* toxin, predominantly synthesized by *Fusarium graminearum* and *Fusarium oxysporum* [1]. DON is commonly encountered in grain crops such as wheat, barley, and corn that have been contaminated with *Fusarium graminearum* and *Fusarium culmorum* [2]. Moreover, DON can also be detected in animal products and processed foods [3]. DON presents a wide array of toxic effects on both human beings and animals [4]. Diverse responses to DON are observed among animals, with pigs being highly susceptible, followed by poultry, while ruminants exhibit comparatively lower sensitivity [5]. More importantly, pigs are favored as translational and clinical study models due to their resemblance to humans in terms of anatomical size and shape, immunology, genome, and physiology [6]. Thus, pigs serve as an excellent model for investigating the adverse effects of DON.

The liver is regarded as one of the primary organs most targeted by DON, as it functions as the main place for DON’s detoxification and metabolism within the body [7]. It has been demonstrated that consumption of DON in either low or high doses can induce chronic or acute hepatic injury [8]. In addition, it has been evidenced that the hepatotoxicity of DON is attributable to the induction of oxidative stress and apoptosis, as confirmed by a study conducted in piglets [9]. Reactive oxygen species (ROS), particularly superoxide, are continuously generated within the mitochondria. However, if excessive superoxide anions are not effectively converted into hydrogen peroxide by superoxide dismutase (SOD), the release of superoxide anions from the mitochondria will cause cellular and at least organ damage, including liver impairment [10,11]. Hence, the liver can be regarded as a substantial focus of DON. Additionally, it serves as a target for nutritional intervention aimed at mitigating the adverse effects caused by DON, with a specific focus on oxidative damage and apoptosis.

Selenium (Se), a significant trace element, exhibits antioxidant capabilities, anti-carcinogenic properties, and detoxification capacities [12]. This element is an essential constituent of selenoproteins such as glutathione peroxidase, thioredoxin reductase, and iodothyronine deiodinase [13]. Selenomethionine (Se-Met) is regarded as one of the organoselenium compounds that exhibit elevated biological functions mediated via selenoproteins, and it exhibits a structural resemblance to methionine, wherein the sulfur element in methionine is replaced by the selenium element [14]. The supplementation of Se-Met has been reported to reduce hydrogen peroxide and lipid peroxides within the body, primarily mediated by selenoproteins such as glutathione peroxidase and thioredoxin reductase [15]. Se functions as an active site within selenoproteins, thus conducting a vital role in their enzymatic activity and subsequently safeguarding cellular membranes and organelles against oxygen-radical-induced damage [13]. Importantly, Se-Met was reported to mitigate oxidative stress induced by DON in the small intestinal epithelium of C57BL/6 mice [16]. Most recently, Se-Met has been demonstrated to exert protective effects for mitigating oxidative stress in poultry through the Nrf2 pathway [17,18,19]. Xie et al. (2023), using an in vitro model of chicken liver cells, demonstrated that Se-Met mitigated hepatic oxidative stress caused by hydrogen peroxide challenge [17]. Li et al. (2024) also found that Se-Met effectively decreased oxidative injury associated with Nrf2 pathway regulation of laying hens challenged with lipopolysaccharide [18]. Dong et al. (2024) reported that Se-Met significantly reduced brain oxidative damage and ferroptosis in chickens challenged with decabromodiphenyl ether, which is linked to the Nrf2/GPX4 pathway [19]. However, limited information is available regarding the protective effects of Se-Met in swine, especially in the context of DON exposure. Furthermore, limited information is available regarding the significant protective role of Se-Met in alleviating the hepatic toxicity and oxidative damage provoked by DON in pigs, given their heightened susceptibility to this mycotoxin.

Therefore, this experiment aimed to test the hypothesis that Se deficiency or Se-Met supplementation could potentially deteriorate or mitigate the hepatotoxicity caused by DON, employing weaned piglets as a research model.

## 2. Materials and Methods

The animal protocols used in this research were authorized by the Institutional Animal Care and Use Committee of Jiangxi Agricultural University (No. JXAULL-20220627).

### 2.1. DON and Se-Met Information

The DON (purity ≥ 98%) and Se-Met (purity ≥ 99%) were acquired from Shanghai Yujing Biotechnology Company (Shanghai, China), and Sigma Aldrich (St. Louis, MO, USA), respectively.

### 2.2. Animals and Experimental Design

Twenty-four healthy weaned piglets (Duroc × Landrace × Yorkshire, with a mean body weight of 6.78 kg and age of 28 days) were randomly assigned to four dietary treatments (n = 6). The four treatments included the control group, which received a dose of 0.3 mg/kg of Se without DON treatment, and the DON treatment groups receiving doses of 0, 0.3, or 0.5 mg/kg Se. The Se source used in this study was Se-Met. A dose of 0.3 mg/kg of Se was supplemented to meet the nutritional requirements of the piglets, while a dosage of 0.5 mg/kg of Se was supplemented to reach the up-limit level of Se in swine feed as set by the EU, the FDA in the United States, and China’s official standard [20]. The supplemental dose of DON was administered at a rate of 3 mg/kg as per the findings documented by Bracarense et al. (2012) [21]. All piglets were raised individually in separate cages (one pig per cage; cage size: 0.8 m in height, 1.5 m in length, and 1.0 m in width) that were equipped with a nipple drinker and a feeder at the Swine Nutrition Base of Jiangxi Agricultural University. This trial spanned 28 days, within which the pigs were supplied with fresh water. The piglets were provided with experimental diets, with daily feed provisions fixed at 320, 400, 500, and 600 g for the duration of 1, 2, 3, and 4 weeks to maintain uniformity and minimize variations in feed consumption. The diets were prepared to meet the piglets’ nutritional needs as specified by the National Research Council (NRC, 2012), with the exception of Se. The ingredient composition and nutritional levels of basal diet are detailed in Table 1.

### 2.3. Sample Collection

After the 28-day feeding trial concluded, following a 12 h fasting period and body weight measurement, blood was sampled from all piglets through the anterior superior vena cava using a sterile syringe. Subsequently, the samples were kept at room temperature for 1 h. The blood samples underwent centrifugation utilizing a centrifuge operating at 4 °C and rotating at a speed of 3000× *g* for 15 min. Afterward, the serum was carefully pipetted into microtubes. The sample microtubes were promptly frozen using liquid nitrogen and then preserved at a temperature of −80 °C for laboratory analysis.

After blood sampling, the piglets were euthanized and subsequently sampled for hepatic samples. Briefly, the piglets were dissected, and then the abdominal cavity was surgically incised along the midline of the abdomen. Subsequently, the entirety of the liver was extracted and its weight was determined. The liver samples were then collected and carefully fixed in a 4% paraformaldehyde solution for the analysis of histopathology and apoptosis using hematoxylin–eosin staining and immunocytochemical staining. Also, liver samples were harvested for the analysis of hepatic antioxidant parameters and ROS level. In addition, liver samples were collected for the analysis of RT-qPCR and Western blotting.

### 2.4. Laboratory Analysis

#### 2.4.1. Hepatic Histopathological Analysis

The liver samples were retrieved from the paraformaldehyde solution and subjected to a series of formal procedures. These included dehydration, transparency enhancement, embedding, sectioning, spreading, and deparaffinization. Subsequently, a staining process using hematoxylin–eosin was administered. The liver histopathology was evaluated utilizing the Images Advanced 3.2 software (Motic, Xiamen, China).

#### 2.4.2. Serum Biochemical Parameters Related to Hepatic Functions

The serum biochemical parameters were quantitatively analyzed by using kits (Nanjing Jiancheng, Nanjing, China), with strict adherence to the provided instructions. The serum biochemical parameters included total bilirubin (TBIL) level, alanine aminotransferase (ALT) activity, aspartate aminotransferase (AST) activity, lactate dehydrogenase (LDH) activity, and alkaline phosphatase (ALP) activity.

#### 2.4.3. ROS Level in The Liver

Following the preparation of frozen sections from liver tissues, these sections were subsequently drawn and stained, and the nuclei were repainted using DAPI. Once sealed, images were captured, with nuclei appearing as blue in the DAPI channel while being red in the positive ROS channel. The observation and capture of images were performed using a Nikon Eclipse C1 fluorescence microscope and Nikon DS-U3 graphics program (Nikon, Tokyo, Japan). Finally, the ROS levels were assessed using Image-J software version 1.53t (NIH, Bethesda, MD, USA).

#### 2.4.4. Hepatic Antioxidant Parameters

The liver tissue was homogenized using a glass homogenizer with ice-cold 0.9% normal saline solution, at a ratio of 1:9 (*w*:*v*). The resulting homogenate was obtained by centrifuging at 12,000× *g*, 4 °C for 15 min. Afterward, the liver tissue homogenate was analyzed for hepatic antioxidant parameters such as total antioxidant capacity (T-AOC), glutathione peroxidase (GSH-Px) activity, glutathione (GSH) level, catalase (CAT) activity, total superoxide dismutase (T-SOD) activity, and malondialdehyde (MDA) level utilizing commercially available kits from Nanjing Jiancheng located in Nanjing, China. Furthermore, the protein content in the liver tissue homogenate was analyzed utilizing a BCA kit (Nanjing Jiancheng, Nanjing, China).

#### 2.4.5. RT-qPCR Analysis

The RT-qPCR analysis was conducted following the method outlined in our previous study [22]. Briefly, approximately 100 mg of each liver tissue was utilized for the extraction of total RNA, employing TRIZOL reagent (TransGen, Beijing, China). Afterward, the total RNA was assessed in terms of quality and concentration, followed by the synthesis of cDNA utilizing a commercial kit (TransGen, Beijing, China). Finally, the RT-qPCR analysis was conducted utilizing the PerfectStart^®^ Uni RT & qPCR kit (TransGen, Beijing, China) on a QuanStudioTM 5 Real-Time PCR Instrument (Thermo Fisher Scientific, Waltham, MA, USA). The primers utilized for RT-qPCR are presented in Table 2. *β-actin* was employed as an internal reference to analyze the relative mRNA levels of the genes using the 2^−ΔΔCt^ method.

#### 2.4.6. Western Blot Analysis

The total protein was extracted from liver samples utilizing a RIPA lysate that contained 1 mM of PMSF and 2 mM of phosphatase inhibitor (Beyotime, Shanghai, China). The protein levels of the extracted samples were analyzed utilizing the BCA assay kit (Jiancheng, Nanjing, China). The proteins were separated using a 10% SDS-PAGE gel (Beyotime, Shanghai, China), and subsequently transferred onto PVDF membrane (Merck Millipore, MA, USA). The membrane was sealed utilizing 5% skimmed milk. Subsequently, it was incubated at 4 °C overnight with a diluted primary antibody (1:1000, Cell Signaling Technology, Boston, FL, USA). The β-actin was utilized as an internal reference in this study. Afterwards, relevant secondary antibodies (1:5000, CST, Boston, MA, USA) were incubated at room temperature for a duration of 2 h. Lastly, the signal was detected utilizing an advanced chemiluminescence system and quantified through the usage of Image J 1.53 t software.

### 2.5. Statistical Analysis

All data were analyzed by one-way analysis of variance, along with Duncan’s multiple range test for conducting multiple comparisons among treatments (SPSS 22.0, Chicago, IL, USA). *p* < 0.05 was regarded as statistical difference, while 0.05 ≤ *p* < 0.10 was regarded as a trend of statistical difference.

## 3. Results

### 3.1. Liver Weight and Histopathology

**Figure 1:**
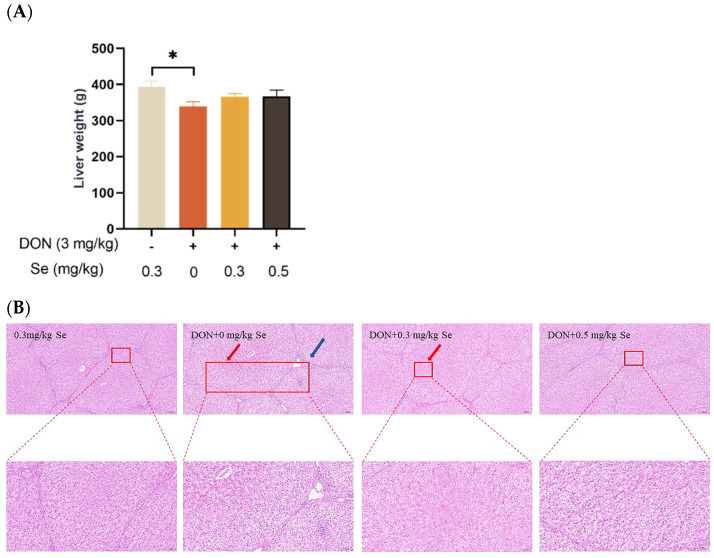
Effects of selenomethionine on liver weight and hepatic histopathology of piglets fed deoxynivalenol (DON)-contaminated diets. (**A**) Liver weight. (**B**) Liver histopathology. Each bar in the graph symbolizes mean ± SEM with 6 replicates. * *p* < 0.05. The red arrow indicates hepatic sludge and the blue arrow indicates inflammatory infiltration.

As shown in Figure 1, compared with the 0.3 mg/kg Se group, the liver weight of the piglets showed no statistical difference in the DON + 0.3 mg/kg Se group (*p* > 0.05), but was reduced in the DON + 0 mg/kg Se group (*p* < 0.05). Regarding liver histopathology, there were no obvious lesions in the livers of piglets in both the 0.3 mg/kg Se group and the DON + 0.5 mg/kg Se group. However, in the DON + 0 mg/kg Se group, there was observed a disordered arrangement of hepatocytes, along with noticeable hepatocellular siltation and inflammatory infiltration. Compared with the DON + 0 mg/kg Se group, a reduced degree of siltation was observed in the liver of piglets in the DON + 0.3 or 0.5 mg/kg Se groups.

### 3.2. Serum Biochemical Parameters Related to Hepatic Functions

**Figure 2:**
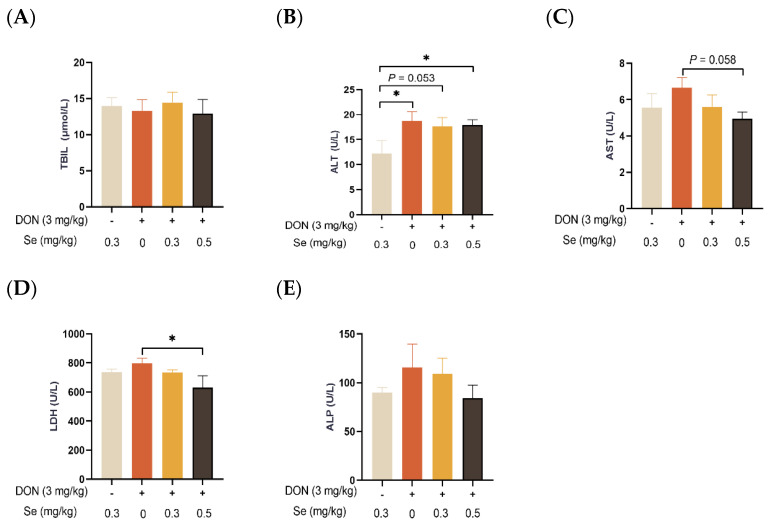
Effects of selenomethionine on serum biochemical parameters related to hepatic functions of piglets fed deoxynivalenol (DON)-contaminated diets. (**A**) TBIL. (**B**) ALT. (**C**) AST. (**D**) LDH. (**E**) ALP. Each bar in the graph symbolizes mean ± SEM with 6 replicates. * *p* < 0.05.

As displayed in Figure 2, the serum TBIL concentration and ALP activity of the piglets were not affected by dietary treatments (*p* > 0.05). In comparison to the 0.3 mg/kg Se without DON treatment group, the ALT activity in the serum of the piglets was observed to be elevated in the DON groups, regardless of the supplemental Se levels (*p* < 0.05 or *p* = 0.053). In the presence of DON exposure, the serum enzymatic activities of AST (*p* = 0.058) and LDH (*p* < 0.05) were decreased when the piglets were administered 0.5 mg/kg Se in place of 0 mg/kg Se.

### 3.3. Hepatic ROS Level

**Figure 3:**
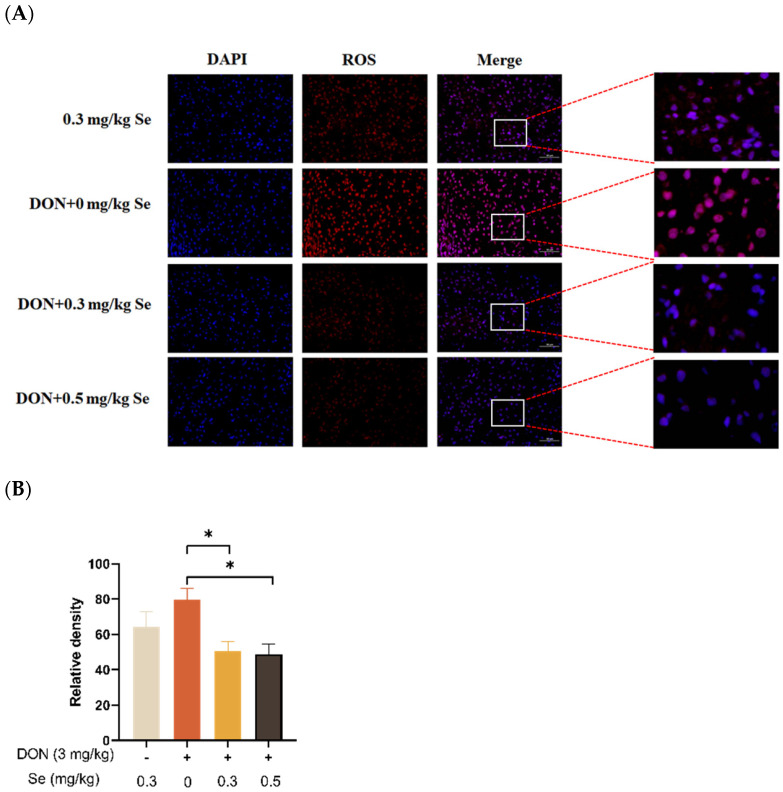
Effects of selenomethionine on hepatic reactive oxygen species (ROS) level of piglets fed deoxynivalenol DON)-contaminated diets. (**A**) Representative ROS-stained paraffin sections. Magnification 400×, scale bar = 50 µm. Dihydroethidium-stained red fluorescence shows ROS level. (**B**) Relative fluorescence density. Each bar in the graph symbolizes mean ± SEM with 6 replicates. * *p* < 0.05.

The effects of selenomethionine on the hepatic ROS level of piglets fed DON-contaminated diets is shown in Figure 3. Compared with the DON + 0 mg/kg Se group, the ROS levels in the liver of the piglets were decreased in the DON + 0.3 or 0.5 mg/kg Se groups (*p* < 0.05).

### 3.4. Mitochondrial Function-Related Genes Expression in Liver

**Figure 4:**
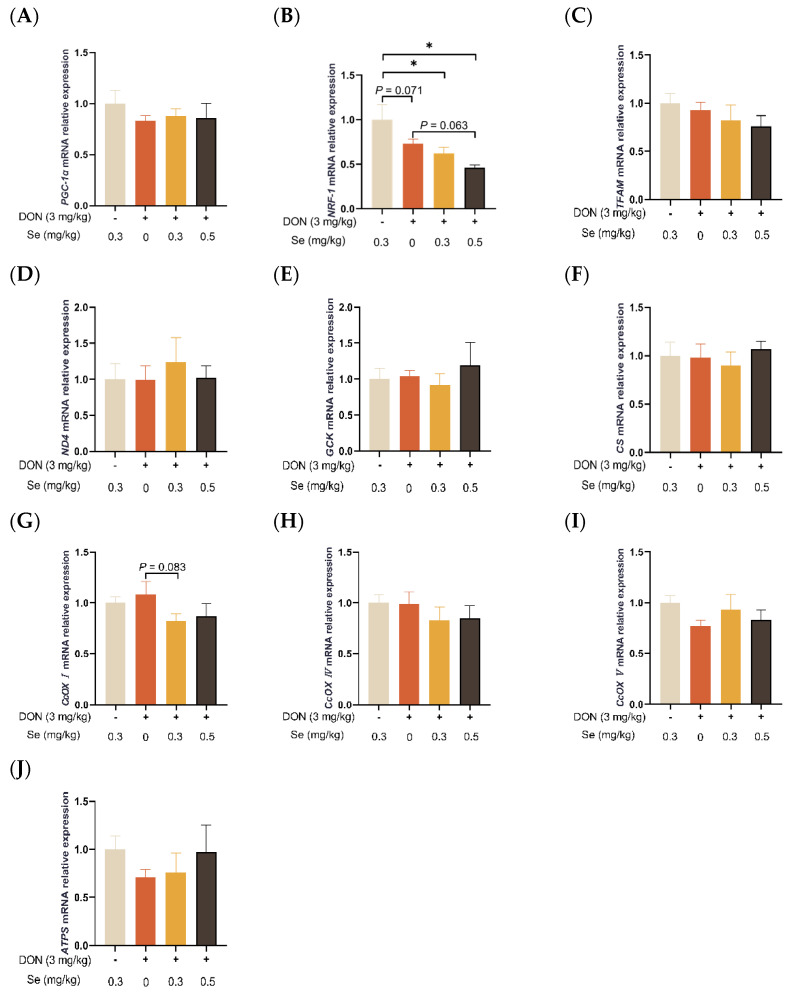
Effects of selenomethionine on hepatic relative mRNA expression of mitochondrial function-related genes in piglets fed deoxynivalenol (DON)-contaminated diets. (**A**) *PGC-1α* mRNA level. (**B**) *NRF*-*1* mRNA level. (**C**) *TFAM* mRNA level. (**D**) *ND4* mRNA level. (**E**) *GCK* mRNA level. (**F**) *CS* mRNA level. (**G**) *CcOX I* mRNA level. (**H**) *CcOX IV* mRNA level. (**I**) *CcOX V* mRNA level. (**J**) *ATPS* mRNA level. Abbreviations: PGC-1α, peroxisome proliferators-activated receptor γ coactivator-1α; NRF-1, nuclear respiratory factor 1; TFAM, mitochondrial transcription factor A; ND4, NADH dehydrogenase subunit 4; GCK, glucokinase; CS, citrate synthase; CcOX I, cytochrome c oxidase I; CcOX IV, cytochrome c oxidase IV; CcOX V, cytochrome c oxidase V; ATPS, adenosine triphosphate synthase. Each bar in the graph symbolizes mean ± SEM with 6 replicates. * *p* < 0.05.

As summarized in Figure 4, the hepatic relative mRNA level of *NRF-1* was down-regulated in the piglets in the DON + 0.3 mg/kg Se group, as compared to the 0.3 mg/kg Se group (*p* < 0.05). However, compared with the DON + 0 mg/kg Se group, the piglets in the DON + 0.5 mg/kg Se group had down-regulated relative mRNA levels of *NFR-1* in their livers (*p* = 0.063). Moreover, compared with the DON + 0 mg/kg Se group, piglets in the DON + 0.3 mg/kg Se group had down-regulated relative mRNA levels of *CcOX I* in the liver (*p* = 0.083).

### 3.5. Apoptosis-Related Genes Expression in Liver

**Figure 5:**
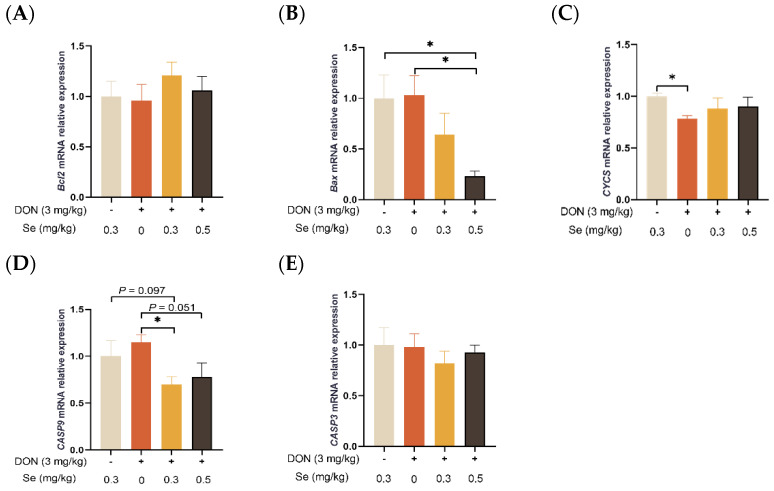
Effects of selenomethionine on hepatic relative mRNA expression of apoptosis-related genes in piglets fed deoxynivalenol (DON)-contaminated diets. (**A**) *Bcl2* mRNA level. (**B**) *Bax* mRNA level. (**C**) *CYCS* mRNA level. (**D**) *CASP9* mRNA level. (**E**) *CASP3* mRNA level. Abbreviations: Bcl2, B-cell lymphoma 2; Bax, Bcl2 associated X; CYCS, cytochrome c; CASP9, caspase 9; CASP3, caspase 3. Each bar in the graph symbolizes mean ± SEM with 6 replicates. * *p* < 0.05.

As shown in Figure 5, the hepatic relative mRNA level of *CASP9* was decreased in piglets in the DON + 0.3 mg/kg Se group, in comparison to the 0.3 mg/kg Se group (*p* = 0.097). Nevertheless, when compared with the DON + 0 mg/kg Se group, the piglets in the DON + 0.5 mg/kg Se group had down-regulated relative mRNA levels of *Bax* (*p* < 0.05) and CASP9 in their livers (*p* = 0.051). Moreover, compared with the DON + 0 mg/kg Se group, piglets in the DON + 0.3 mg/kg Se group had down-regulated relative mRNA levels of *CASP9* in the liver (*p* < 0.05).

### 3.6. Hepatic Antioxidant Capacity

**Figure 6:**
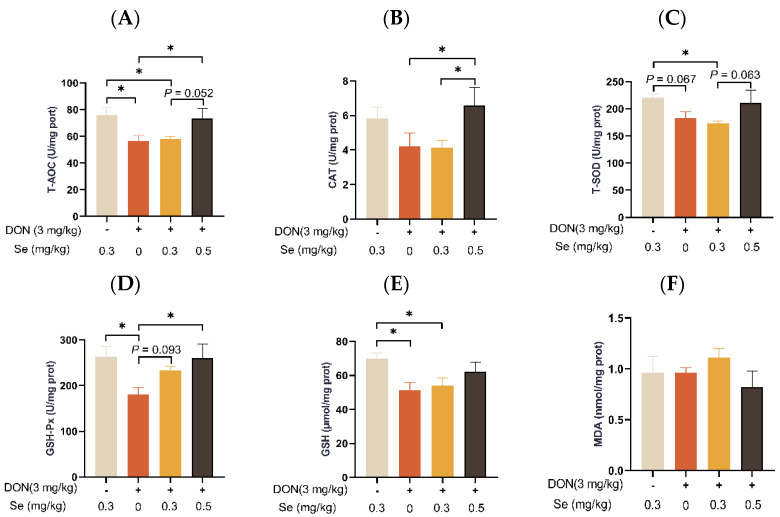
Effects of selenomethionine on hepatic antioxidant status in piglets fed deoxynivalenol (DON)-contaminated diets. (**A**) T-AOC. (**B**) CAT. (**C**) T-SOD. (**D**) GSH-Px. (**E**) GSH. (**F**) MDA. Each bar in the graph symbolizes mean ± SEM with 6 replicates. * *p* < 0.05.

The MDA level in the livers of the piglets was unaffected by dietary treatments (*p* > 0.05) (Figure 6). Compared to the 0.3 mg/kg Se group, the T-AOC, T-SOD activity, and GSH content in the livers of the piglets were decreased in the DON + 0.3 mg/kg Se group (*p* < 0.05). However, when under DON conditions, the livers of piglets in the 0.5 mg/kg Se group exhibited the highest antioxidant capacity among the 0, 0.3, and 0.5 mg/kg Se groups. Specifically, when compared to the DON + 0 mg/kg Se group, the piglets in the DON + 0.5 mg/kg Se group had elevated T-AOC, CAT activity, and GSH-Px activity in their livers (*p* < 0.05). Moreover, compared with the DON + 0 mg/kg Se group, piglets in the DON + 0.3 mg/kg Se group had elevated GSH-Px activity in the liver (*p* < 0.05).

### 3.7. Antioxidant-Related Genes Expression in the Liver

**Figure 7:**
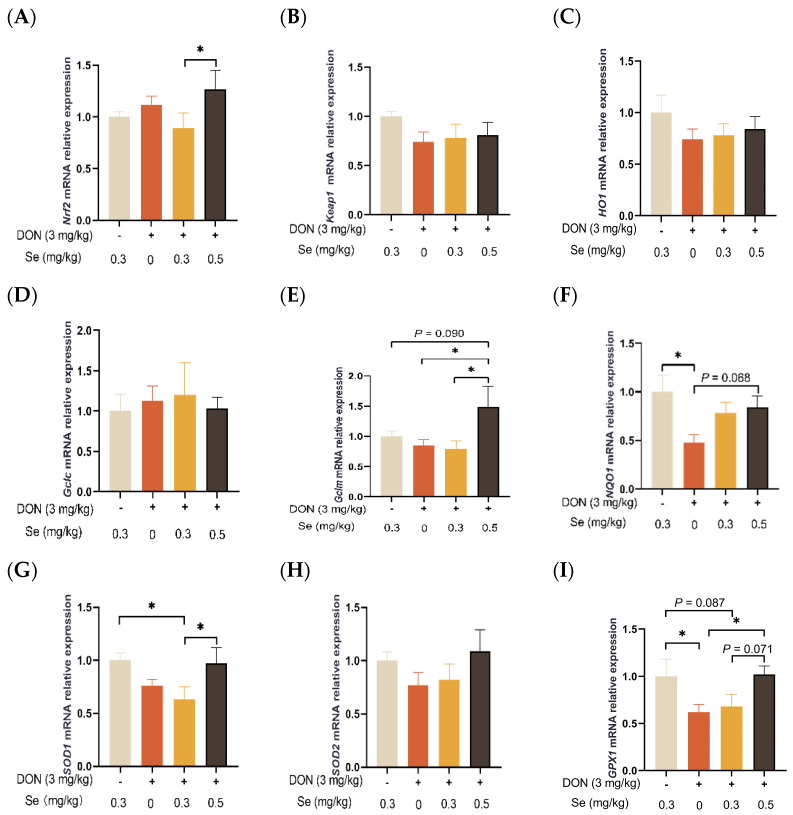
Effects of selenomethionine on hepatic relative mRNA expression of antioxidant-related genes in piglets fed deoxynivalenol (DON)-contaminated diets. (**A**) *Nrf2* mRNA level. (**B**) *Keap1* mRNA level. (**C**) *HO1* mRNA level. (**D**) *Gclc* mRNA level. (**E**) *Gclm* mRNA level. (**F**) *NQO1* mRNA level. (**G**) *SOD1* mRNA level. (**H**) *SOD2* mRNA level. (**I**) *GPX1* mRNA level. Abbreviations: Nrf2, nuclear factor erythroid 2-related factor 2; Keap1, Kelch-like ECH-associated protein l; HO1, heme oxygenase 1; Gclc, glutamate-cysteine ligase catalytic subunit; Gclm, glutamate-cysteine ligase modifier subunit; NQO1, NAD(P)H quinone oxidoreductase 1; SOD1, superoxide dismutase 1; SOD2, superoxide dismutase 2; GPX1, glutathione peroxidase 1. Each bar in the graph symbolizes mean ± SEM with 6 replicates. * *p* < 0.05.

As shown in Figure 7, the hepatic relative mRNA levels of *Keap1*, *HO1*, *GCLC*, and *SOD2* in the piglets were not affected by the dietary treatments (*p* > 0.05). The relative mRNA levels of *SOD1* (*p* < 0.05) and *GPX1* (*p* = 0.087) were found to be down-regulated in the livers of piglets in the DON + 0.3 mg/kg Se group when compared to the 0.3 mg/kg Se group. However, when under DON conditions, the livers of piglets in the 0.5 mg/kg Se group exhibited the highest relative mRNA level of antioxidant-related genes among the 0, 0.3, and 0.5 mg/kg Se groups. Specifically, compared with the DON + 0 mg/kg Se group, piglets in the DON + 0.5 mg/kg Se group had up-regulated relative mRNA levels of *Gclm* (*p* < 0.05), *NQO1* (*p* = 0.068), and *GPX1* (*p* < 0.05) in their livers. Moreover, compared with the DON + 0.3 mg/kg Se group, piglets in the DON + 0.5 mg/kg Se group had up-regulated relative mRNA levels of *Nrf2* (*p* < 0.05), *Gclm* (*p* < 0.05), *SOD1* (*p* < 0.05), and *GPX1* (*p* = 0.071) in the liver.

### 3.8. Protein Expression of Key Proteins in MAPK Pathways

**Figure 8:**
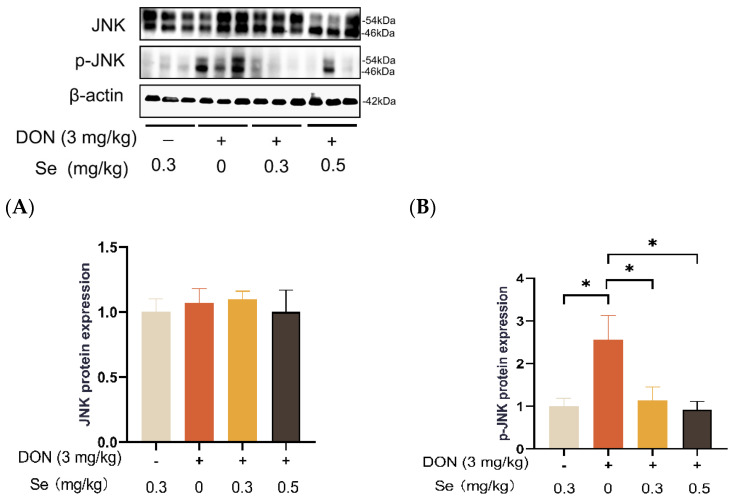
Effects of selenomethionine on hepatic relative protein expression of JNK and p-JNK of piglets fed deoxynivalenol (DON)-contaminated diets. (**A**) JNK protein level. (**B**) p-JNK protein level. Abbreviations: JNK, c-Jun N-terminal kinase; p-JNK, phosphorylated JNK. Each bar in the graph symbolizes mean ± SEM with 6 replicates. * *p* < 0.05.

**Figure 9:**
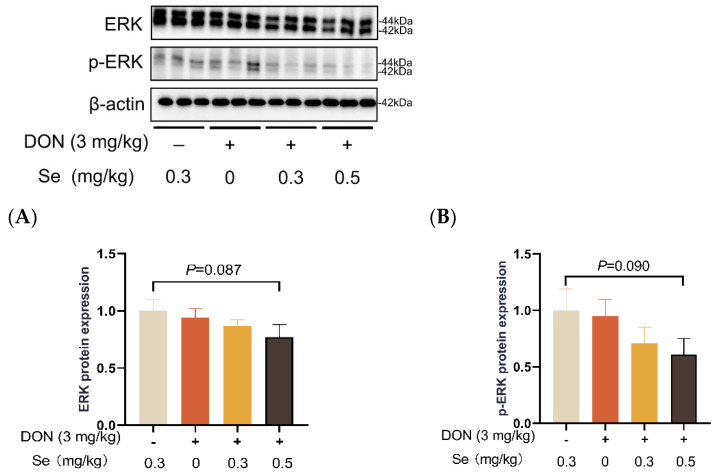
Effects of selenomethionine on hepatic relative protein expression of ERK and p-ERK in piglets fed deoxynivalenol (DON)-contaminated diets. (**A**) ERK protein level. (**B**) p-ERK protein level. Abbreviations: ERK, extracellular regulated protein kinases; p-ERK, phosphorylated ERK. Each bar in the graph symbolizes mean ± SEM with 6 replicates. * *p* < 0.05.

**Figure 10:**
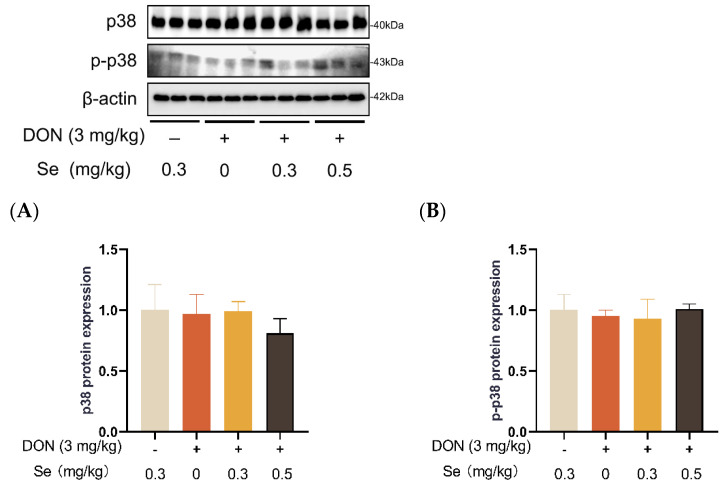
Effects of selenomethionine on hepatic relative protein expression of p38 and p-p38 in piglets fed deoxynivalenol (DON)-contaminated diets. (**A**) p38 protein level. (**B**) p-p38 protein level. Abbreviations: p38, p38 mitogen activated protein kinases; p-p38, phosphorylated p38. Each bar in the graph symbolizes mean ± SEM with 6 replicates. * *p* < 0.05.

Compared to the 0.3 mg/kg Se group, the protein expression level of p-JNK was increased in the livers of the piglets in the DON + 0 mg/kg Se group (*p* < 0.05). However, compared with the DON + 0 mg/kg Se group, the protein expression level of p-JNK was down-regulated in the livers of piglets in both the DON + 0.3 mg/kg Se group and the DON + 0.5 mg/kg Se group (*p* < 0.05) (Figure 8). In addition, compared with the 0.3 mg/kg Se group, the protein expression levels of ERK (*p* = 0.087) and p-ERK (*p* = 0.090) tended to be down-regulated in the DON + 0.5 mg/kg Se group (Figure 9). However, the protein expression levels of p38 and p-p38 were not impacted by the dietary treatments (*p* > 0.05) (Figure 10).

## 4. Discussion

There is observed variability among animals regarding their sensitivity to DON, with pigs exhibiting a high susceptibility to this mycotoxin [23]. The liver holds significant importance as a toxic target for DON owing to its crucial role in metabolism and detoxification processes [13]. Furthermore, it acts as a target for nutritional intervention endeavors focused on alleviating the adverse effects of DON exposure, particularly emphasizing oxidative damage and apoptosis. It has been demonstrated that Se-Met administration reduced oxidative stress by reducing hydrogen peroxide and lipid peroxidation and inhibiting excessive ROS production [12,16,24]. Hence, this research aimed to explore the influence of Se-Met on hepatic injury, oxidative stress, mitochondrial function, and apoptosis of piglets fed deoxynivalenol-contaminated diets. In this study, compared with the 0.3 mg/kg Se group, the liver weight of the piglets was decreased in the DON + 0 mg/kg Se group. This result suggests that the exposure to DON resulted in detrimental impacts on the liver weight of the piglets, particularly in cases where there was a deficiency of Se in the diets. Similarly, Awad et al. (2006) observed that absolute liver weight was notably decreased in broiler chickens fed DON-contaminated diets for 21 days [25]. Similarly, Lucke et al. (2017) reported that DON markedly reduced absolute and relative liver weights of broiler chickens in a 3-week feeding trial [26]. According to Peng et al. (2017), the reduction in liver weight is likely attributed to protein synthesis inhibition and inflammation caused by DON exposure [11]. Accordingly, in the DON + 0 mg/kg Se group, there was observed a disordered arrangement of hepatocytes, along with noticeable hepatocellular siltation and inflammatory infiltration. In alignment with our findings, previous studies have documented that DON-induced liver inflammation is characterized by dilated central lobe veins, hepatic medulla disease, and inflammatory cell infiltration [8,27]. Compared with the DON + 0 mg/kg Se group, a reduced degree of siltation was observed in the livers of piglets in the DON + 0.3 mg/kg Se group and the DON + 0.5 mg/kg Se group. Those results suggest that 0.5 mg/kg Se supplementation could improve the liver histopathology in piglets fed DON-contaminated diets.

Next, we measured the serum biochemical parameters related to the hepatic functions of piglets. Previous studies have reported a significant increase in the enzymatic activities of ALT, AST, and LDH in piglets fed with DON-contaminated diets [9] and in mice orally administered with DON [28]. In the present study, in comparison to the 0.3 mg/kg Se without DON treatment group, the ALT activity in the serum of the piglets was observed to be elevated in the DON groups, regardless of the supplemental Se levels. ALT and AST are hepatocyte enzymes located intracellularly, and liver damage leads to elevations in serum ALT and AST activities [29]. This finding suggests that DON caused hepatic dysfunction. Serum LDH activity is also a key biomarker of liver injury [30]. In the current study, in the presence of DON exposure, the enzymatic activities of AST and LDH in the serum of the piglets were observed to decrease when the piglets were administered 0.5 mg/kg Se instead of 0 mg/kg Se. Those results indicate that Se-Met supplementation improved the liver function of the piglets subjected to DON exposure.

The overproduction of ROS is associated with oxidative stress and has the potential to cause damage to proteins, lipids, and DNA [31]. The liver is particularly susceptible to damage from ROS, and the oxidative stress caused by ROS is frequently linked to hepatic disorders [32]. It has been reported that the accumulation of ROS caused by DON is of significant relevance in the early-stage liver injury observed in mice [33]. Furthermore, the piglets that were exposed to DON exhibited a substantial rise in ROS production within their hepatocytes [9]. In the present study, compared with the DON + 0 mg/kg Se group, the ROS level in the livers of the piglets was decreased in both the DON + 0.3 mg/kg Se group and the DON + 0.5 mg/kg Se group. Those findings suggest that 0.5 mg/kg Se as Se-Met could effectively mitigate the oxidative stress in the livers of piglets under DON exposure.

Given that ROS are generated in the internal mitochondrial respiratory system and mitochondria is a potential target of DON, there exists a close relationship between DON-induced oxidative stress and mitochondrial dysfunction [10]. The SOD2 is a biomarker for assessing mitochondrial function. This enzyme is located within the mitochondrial matrix, facilitating the conversion of superoxide into the less reactive hydrogen peroxide. Moreover, this conversion of hydrogen peroxide aids in the passive diffusion process away from the mitochondrial matrix, effectively preventing a substantial accumulation of superoxide in close proximity to the ATP-generating site [34]. In the present experiment, the hepatic *SOD2* mRNA level in the piglets was unaffected by dietary treatments. In addition, PGC-1α is an essential regulator of mitochondrial biogenesis [35]. Following PGC-1α activation, NRF-1 and NRF-2 and TFAM are subsequently activated [32]. It has been documented that trichlorfon exposure induced oxidative stress and aberrant mitochondrial biogenesis in the jejunum of piglets, which is characterized by decreased expression of *PGC-1α*, *NRF-1*, and *TFAM* [36]. Similarly, DON exposure has been reported to have decreased hepatic mRNA expression of *TFAM* and *PGC-1α* in piglets [9]. In the present study, the relative mRNA level of *NRF-1* was down-regulated in the livers of the piglets in the DON + 0.3 mg/kg Se group, as compared to the 0.3 mg/kg Se group. However, compared with the DON + 0 mg/kg Se group, the piglets in the DON + 0.5 mg/kg Se group had down-regulated relative mRNA levels of *NFR-1* in their livers. Those findings indicate that 0.5 mg/kg Se as Se-Met could mitigate mitochondrial dysfunction in the liver of piglets under DON exposure.

It has also been demonstrated that DON exposure caused cell apoptosis by promoting ROS overproduction in porcine intestinal epithelial cells [37]. Here, we examined hepatic apoptosis by analyzing the apoptosis-related gene expression in the livers of piglets. We found that the relative mRNA level of *CASP9* was down-regulated in the livers of piglets in the DON + 0.3 mg/kg Se group, in comparison to the 0.3 mg/kg Se group. These results provide evidence that exposure to DON could cause hepatic apoptosis in piglets, despite being in conditions of dietary Se sufficiency (0.3 mg/kg). It has been reported that apoptosis induced by DON occurs through a mechanism dependent on mitochondrial cysteine asparaginase [38]. Similarly, Bensassi et al. (2012) have found that exposure to DON increased the permeability of the mitochondrial membrane, consequently facilitating the release of CYCS into the cytoplasm [21]. This, in turn, activated CASP9 and led to the subsequent activation of CASP3, thereby inducing apoptosis [21]. In the process of apoptosis, Bcl-2 and Bax participate in a cascade reaction, resulting in the release of cytochrome c and caspases activation, ultimately leading to cell death [39]. However, under DON exposure conditions, the mRNA level of *CASP9* was further up-regulated in the livers of piglets fed 0 vs. 0.3 mg/kg Se diets in the present study. This suggests that Se deficiency could aggravate hepatic apoptosis induced by DON exposure. Importantly, when compared with the DON + 0 mg/kg Se group, the piglets in the DON + 0.5 mg/kg Se group had down-regulated relative mRNA levels of *Bax* and *CASP9* in their livers. Consistently, Se-Met has recently been reported to reduce Cadmium tellurium quantum dots (CdTe QDs)-induced apoptosis in hepatocytes while preserving redox balance and mitochondrial function [40]. Therefore, increasing the Se supply to the up-limited dosage (0.5 mg/kg) using Se-Met could mitigate hepatic apoptosis in piglets fed DON-contaminated diets.

Se functions as an active site within selenoproteins [13]. There are 25 identified selenoproteins in pigs, in which several antioxidant selenoproteins (such as glutathione peroxidase family and thioredoxin reductase family selenoproteins) could safeguard cellular membranes and organelles against oxygen-radical-induced damage [41]. Here, we further determined the antioxidant status in the livers of the piglets. The biological antioxidant system comprises a range of enzymes, including SOD, GSH-Px, and CAT, which provide protection against oxidative stress [32]. The SOD converts free radicals from superoxide anions into hydrogen peroxide, subsequently decomposed by CAT and GSH-Px, thereby safeguarding cells against oxidative damage [39]. In the present study, compared to the 0.3 mg/kg Se group, the T-AOC, T-SOD activity, and GSH level in the livers of the piglets were decreased in the DON + 0.3 mg/kg Se group. Previous studies have also reported decreased activities of T-SOD in serum and liver after DON exposure in piglets [9], further supporting our results. However, when under DON conditions, the livers of piglets in the 0.5 mg/kg Se group exhibited the highest antioxidant capacity among the 0, 0.3, and 0.5 mg/kg Se groups. When compared to the DON + 0 mg/kg Se group, the piglets in the DON + 0.5 mg/kg Se group had elevated T-AOC, CAT activity, and GSH-Px activity in their livers. Those findings indicate that 0.5 mg/kg Se as Se-Met could effectively enhance the hepatic antioxidant capacity of piglets under DON exposure.

Nrf2 plays a critical role as a transcriptional factor regulating its downstream antioxidant genes and makes valuable contributions to antioxidant defense mechanisms [42]. The Nrf2/Keap1 pathway possesses the ability to regulate more than 100 downstream genes, focusing particularly on genes related to redox signaling, oxidative stress, and cell protection target genes [43]. Herein, we determined the gene expressions of Nrf2/Keap1 and its downstream antioxidant genes, including *Nrf2*, *Keap1*, *HO1*, *Gclc*, *Gclm*, *NQO1*, *SOD1*, *SOD2*, and *GPX1*. In the present experiment, the relative *mRNA* levels of *SOD1* and *GPX1* were found to be down-regulated in the livers of piglets in the DON + 0.3 mg/kg Se group when compared to the 0.3 mg/kg Se group. The aforementioned findings suggest that exposure to DON led to a decline in the antioxidant defense within the livers of the piglets, despite them being fed diets that contained sufficient Se (0.3 mg/kg). However, when under DON conditions, the livers of piglets in the 0.5 mg/kg Se group exhibited the highest relative mRNA level of antioxidant-related genes among the 0, 0.3, and 0.5 mg/kg Se groups. Specifically, compared to the DON + 0 mg/kg Se group, piglets in the DON + 0.5 mg/kg Se group had up-regulated relative mRNA levels of *Gclm*, *NQO1*, and *GPX1* in their livers. Moreover, compared with the DON + 0.3 mg/kg Se group, piglets in the DON + 0.5 mg/kg Se group had up-regulated relative mRNA levels of *Nrf2*, *Gclm*, *SOD1*, and *GPX1* in the livers. These findings are consistent with a study demonstrating that Se supplementation activated the Nrf2 signaling pathway to attenuate hepatotoxicity induced by hexavalent chromium [44]. Given these findings, the improved antioxidant capacity with 0.5 mg/kg Se supplementation can be attributed to the regulation of the hepatic Nrf2/Keap1 and its downstream antioxidant genes in piglets that were fed diets contaminated with DON.

Mitogen-activated protein kinases (MAPKs, i.e., JNK, p38 and ERK) are serine/threonine kinases that play crucial roles in responding to various internal and external stimuli [45]. More importantly, the underlying mechanism of DON exposure toxicity has been associated with the selective activation of MAPKs pathways [46]. Accordingly, we further analyzed the MAPKs expression in the livers of piglets. Compared to the 0.3 mg/kg Se group, the protein expression abundance of p-JNK was elevated in the livers of piglets in the DON + 0 mg/kg Se group. It has been found that DON binds to the 80S subunit found in ribosomes within eukaryotic cells. This binding process activates the MAPK pathway, leading to the initiation of an oxidative stress response. Consequently, the synthesis of proteins and DNA becomes obstructed, resulting in the generation of cytotoxic and immunotoxic effects [1,47]. However, compared with the DON + 0 mg/kg Se group, the protein expression level of p-JNK was down-regulated in the livers of piglets in both the DON + 0.3 mg/kg Se group and the DON + 0.5 mg/kg Se group. The aforementioned findings indicate that the inhibition of JNK MAPK by Se-Met is an underlying mechanism contributing to the protective effects observed in the livers of piglets that were fed diets contaminated with DON.

## 5. Conclusions

In conclusion, Se-Met supplementation mitigated liver dysfunction, oxidative injury, and apoptosis through enhancing antioxidant capacity and inhibiting the JNK MAPK pathway in piglets fed DON-contaminated diets.

## Figures and Tables

**Table 1:** Ingredient composition and nutritional levels of basal diet.

Item	Ingredient, %	Item	Nutrient Levels ^c^
Corn	36.50	Digestible energy, kcal/kg	3467.00
Extruded corn	20.00	Crude protein, %	19.07
Extruded soybean	10.00	Lysine, %	1.39
Soybean meal	6.50	Methionine, %	0.48
Fish meal	5.00	Threonine, %	0.79
Whey powder	5.00	Calcium, %	0.68
Soy protein concentrate	4.00	Total phosphorus, %	0.55
Wheat flour	5.00	Available phosphorus, %	0.33
Wheat bran	4.00		
Limestone	0.70		
Dicalcium phosphate	0.50		
Salt	0.30		
Chromium trioxide	0.25		
Choline chloride (60%)	0.10		
L-Lysine hydrochloride	0.50		
DL-Methionine	0.16		
L-Threonine	0.10		
L-Tryptophan	0.03		
L-Valine	0.14		
Vitamin premix ^a^	0.16		
Mineral premix ^b^	0.10		
Zeolite powder	0.96		
Total	100.00		

^a^ The vitamin premix offers the subsequent values per kilogram of diet: vitamin A 10,000 IU, vitamin D_3_, 1500 IU, vitamin E 50 IU, vitamin K_3_ 2.5 mg, vitamin B_1_ 4.5 mg, vitamin B_2_ 12 mg, vitamin B_6_ 10 mg, vitamin B_12_ 60 μg, niacin 60 mg, pantothenic acid 36 mg, folic acid 1 mg, biotin 0.5 mg. ^b^ The mineral premix offers the subsequent values per kilogram of diet: Fe 100 mg, Cu 6 mg, Mn 4 mg, Zn 100 mg, I 0.14 mg. ^c^ Calculated values.

**Table 2:** Primer information for RT-qPCR.

Genes	Accession Number	Primer Sequence (5′-3′)
*NQO1*	NM_001159613.1	F: GCCCAGATATTGTGGCCGAA R: AACTCCCCTATGAGCACACG
*SOD1*	NM_001190422.1	F: AAGGCCGTGTGTGTGCTGAA R: GATCACCTTCAGCCAGTCCTTT
*SOD2*	NM_214127.2	F: GGCCTACGTGAACAACCTGA R: TGATTGATGTGGCCTCCACC
*GPX1*	NM_214201.1	F: TGAATGGCGCAAATGCTCAC R: ATTGCGACACACTGGAGACC
*Keap1*	NM_001114671.1	F: GCCTCATCGAGTTCGCTTAC R: ACGGACCACACTGTCAATCT
*Nrf2*	XM_021075133.1	F: CCCATTCACAAAAGACAAACATTC R: GCTTTTGCCCTTAGCTCATCTC
*HO1*	NM_001004027.1	F: TCAAGCAGAGGGTCCTCGAA R: CCTCTTGCGGATGTCGGATG
*Gclm*	XM_001926378.4	F: GATGCCGCCCGATTTAACTG R: ACAATGACCGAGTACCGCAG
*Gclc*	XM_021098556.1	F: GACGACGCCAATGAGTCTGA R: AGCACCACAAACACCACGTA
*PGC-1α*	NM_213963.2	F: GATGACCCTCCTCACACCAA R: TTGGAGGTGCACTTGTCTCT
*NRF-1*	XM_021078993.1	F: GCTGTGGCAACAGGAAAGAA R: AAGACAGGGTTGGGTTTGGA
*TFAM*	NM_001130211.1	F: GTCTGAAGAGTTGCTTGCGA R: ACCCGTAGACAAAGCACTGA
*GCK*	XM_013985832.2	F: ACCTTCTCCTTTCCCGTGAG R: CCCACGATGTTGTTCCCTTC
*CS*	XM_021091147.1	F: TCAGGAAGTGCTTGTTTGGC R: GGCAGGTGTTTCAGAGCAAA
*ND4*	NM_001097468.2	F: TTATTGGTGCCGGAGGTACTG R: CCCAGTTTATTCCAGGGTTCTG
*CcOX I*	AJ950517.1	F: ATTATCCTGACGCATACACAGCA R: GCAGATACTTCTCGTTTTGATGC
*CcOX IV*	AK233334.1	F: CCAAGTGGGACTACGACAAGAAC R: CCTGCTCGTTTATTAGCACTGG
*CcOX V*	NM_001007517.1	F: ATCTGGAGGTGGTGTTCCTACTG R: GTTGGTGATGGAGGGGACTAAA
*ATPS*	AK230503	F: TGTCCTCCTCCCTATCACACATT R: TAGTGGTTATGACGTTGGCTTGA
*Bcl2*	XM_005666256.3	F: CTTCTGCAAATCCCGGACTG R: CCGTAGGAATCCCAACCAGA
*Bax*	XM_013998624.2	F: TCGCTCACCATCTGGAAGAA R: ATTGTCCTCCGAGACCACTC
*CYCS*	NM_001129970.1	F: ACCTCCATGGTCTCTTTGGG R: TGCCTTTGTTCTTGTTGGCA
*CASP9*	XM_003127618.4	F: TGGAACTCAAGCCAGAGGAG R: CTGCATTCAGGACGTAAGCC
*CASP3*	NM_214131.1	F: CGGACAGTGGGACTGAAGAT R: GCTGCACAAAGTGACTGGAT

## Data Availability

Data are contained within the article.
